# The future of universal health coverage: How can low- and middle-income countries ‘break free from cocoons and transform’?

**DOI:** 10.7189/jogh.14.03021

**Published:** 2024-03-15

**Authors:** Jianzhong Yin, Rui Deng, Qiong Meng, Yuemei Feng, Teng Zhang

**Affiliations:** 1 Yunnan Provincial Key Laboratory of Public Health and Biosafety and School of Public Health, Kunming Medical University, Kunming, China; 2 Baoshan College of Traditional Chinese Medicine, Baoshan, China; 3 Healthy Yunnan Development Think Tank, Kunming, China

Universal health coverage (UHC) is a paramount concern within the health domain. A worldwide consensus exists on attaining UHC by 2030, aiming to improve health equity and enhance universal health [1,2]. However, the global ongoing pursuit of UHC, particularly in low- and middle-income countries, is confronted by several persistent issues and challenges.

There are evident regional and population health disparities. Low- and middle-income countries comprise four-fifths of the global population, creating a substantial demand for health services [3,4]. However, significant imbalances persist across different regions and populations in the distribution, accessibility, and quality of health service resources. This is compounded by low- and middle-income populations often lacking essential social security [5]. Additionally, rural residents, impoverished people, floating populations, racial/ethnic minorities, and other demographic groups are experiencing severe health vulnerability. The risk of falling into and worsening poverty due to illness is substantial. Thus, addressing poverty and disease effectively is imperative [6–8].

Further, the foundation of sustainable development strategies is fragile. There needs to be more collaboration and synergy among different nations. Targeted inter-country exchanges on UHC policies, sharing of best practices and strategies, and cooperation mechanisms are insufficient. The promotion of UHC relies solely on the robustness of a country’s health system, and the fragmentation of health services can easily lead to intermittent service provision across borders, thereby impeding the sustainable development of UHC [9]. In addition, low- and middle-income countries have weaknesses in their services for universal health. For instance, psychological and mental health services, responses to public health emergencies, early childhood development and nutritional improvement, sanitary facilities and residential environment enhancement, and rehabilitation services for people living with disabilities exist [10,11].

Moreover, urgent attention is required to address the health requirements of vulnerable groups. Notably, the mental health service requirements for children and adolescents are increasingly prominent, which have been a crucial factor influencing their healthy development [12,13]. In the context of responding to public health emergencies, mental health services, a vital service for ensuring comprehensive health, still encounter significant gaps in both public awareness and service delivery. People living with schizophrenia undergoing prolonged hospitalisations are facing a deficit in family care and social support, and the ‘from hospital to family to community’ linkage care mode demands immediate consideration and exploration [14]. Additionally, clinicians are confronting overwhelming occupational stress and a dearth of humanistic care and concern for their health.

Furthermore, the impacts of globalisation and population migration underscore an inadequacy of humanistic care for vulnerable groups. For instance, transnational labourers lack access to medical insurance and primary health care services. Linguistic minorities are encountering challenges in obtaining essential health information and services. Populations experiencing famine exposure within the food security framework face lasting effects on the risk of chronic diseases [15–20].

Hence, we recommend the following. First, it is crucial to promote health equity across diverse regions and populations, which involves several things. It is important to rationalise the allocation of health care resources and augment government investment in preventive health care services. For instance, China has steadily increased financial subsidies for basic public health service programs. The per capita cost has escalated from 15 Chinese Yuan in 2009 to 89 in 2023 [21]. Thailand’s general government health expenditure has shown an upward trend, accompanied by the establishment of the Local Health Promotion Fund, which invests an additional 40 Thai Baht per capita to improve health promotion and disease prevention [22]. Further, it is essential to develop a comprehensive strategy for health poverty alleviation. This approach ensures that impoverished people can afford essential health care services and strives to mitigate their disease risks, enhance population quality, and break the vicious cycle of ‘poverty and disease’. Rwanda’s experience demonstrates that the provision of public financial assistance plays a pivotal role in effectively extending health insurance coverage to the extremely poor, vulnerable, and marginalised populations, thereby contributing to the country’s successful attainment of universal health coverage [23]. Moreover, it is important to establish a mechanism to promote the equitable integration of urban and rural health development, with an emphasis on making health promotion in rural communities the focal point of UHC efforts and strengthening the network of community medical and health services. China, for instance, has expanded telemedicine coverage to all impoverished counties and has been extending it to township hospitals in terms of accessibility of health care services [24]. Thailand has expanded the Universal Coverage Scheme to encompass a broader demographic and augmented investments in rural health care facilities, with the objective of enhancing health care services in rural areas [25]. Also, there is a need to deploy medical and health care human resources trained in higher medical colleges and universities to grassroots levels to improve the accessibility and equilibrium of health care service capacity. For example, the provision of rural health care services in Iran has been significantly enhanced through the implementation of a policy requiring medical students to fulfil their compulsory military service period in rural areas [26].

Second, it is important to strengthen the strategic basis of priority development. The strategic position of UHC should be prioritised and UHC should be reintegrated into the global agenda [27]. This emphasises the concept, ‘community of shared future for human health,’ thereby advancing global health governance with an unwavering commitment to the health problems of every individual, regardless of their geographic location. Tangible commitments for concrete action are compulsory at the national level, accompanied by a robust mechanism ensuring universal health equity. For instance, China has effectively implemented the comprehensive ‘Healthy China 2030’ framework to promote UHC. Moreover, the Chinese government has launched initiatives including establishing national sanitary cities and the patriotic health ‘seven special campaigns’ [28,29]. Efforts should be made to facilitate the experience sharing of UHC policies and best practices that is adaptable for reference among nations. Further, stimulating support for research in emerging domains, including global climate risks and the health implications of immigration is essential. This encompasses the following: i) the health equity of new migrants transitioning from rural to urban areas which represented the resettled mobile population in the context of the new urbanisation trends, ii) health discrepancies among elderly populations between urban and rural areas in the context of urban-rural integration development, iii) disparities in home care accessibility for elderly individuals in rural areas, iv) global health issues related to ethnic and racial equality, and v) factors affecting the resilience of low- to middle-income family development.

Third, there should be a focus on improving the health requirements for vulnerable populations. The global coronavirus pandemic has unveiled the fragility of the current global health system. With the distinctive health requirements of vulnerable populations increasingly gaining prominence, we should advocate for the health and health equity of previously neglected regions and vulnerable groups [30,31]. It is necessary to facilitate global collaboration on adolescent health problems. This includes improving the psychosocial impact triggered by adolescent internet addiction, adopting humanistic care to alleviate adolescent anxiety about development, promoting health equity and implementing resilient measures for families with vulnerable children in impoverished areas on the perspective of population migration, safeguarding the health of left-behind children receiving caregiving across generations in rural areas, mitigating social exclusion and discrimination of the offspring of workers in urban emerging industries, and optimising security policies of health equity for children in difficulty. It is important to enhance the life quality for caregivers of people living with disabilities, including people living with psychiatric disorders. Establishing an effective community-based support model is crucial to facilitate people living with psychiatric disorders return to their families and society, fostering their journey towards rehabilitation and social reintegration. The allocation of health care spending in Iran dedicates approximately 3% to mental health services, while a proposed socio-psychological health service model has been implemented as a pilot program across eight cities in the country. This initiative aims to provide comprehensive health care services and improve the overall quality of life for individuals with mental disorders [32]. Moreover, there is a need to address health impacts and support low-income populations in rural areas, as well as elderly workers in urban emerging industries, in the context of equal opportunities. Furthermore, there should be a focus on the life quality for elderly people living with disabilities and empty-nested elderly people, and addressing challenges of digitalisation and social alienation resulting from the digital era, which has contributed to digital disability and health inequalities troubling elderly people. Finally, establishing a global food security network to mitigate long-term health losses and disease risks associated with famine exposure is needed.
